# Complete Genome Assemblies of the Rare Salmonella enterica Serovar Adjame Using Nanopore and Illumina Sequence Reads

**DOI:** 10.1128/MRA.00280-20

**Published:** 2020-08-27

**Authors:** Ruimin Gao, Marc-Olivier Duceppe, Sohail Naushad, Marie Anne Chattaway, Dele Ogunremi

**Affiliations:** a Ottawa Laboratory Fallowfield, Canadian Food Inspection Agency, Ottawa, Ontario, Canada; b Gastrointestinal Bacteria Reference Unit, Public Heath England, London, United Kingdom; University of Maryland School of Medicine

## Abstract

The complete genome sequences of 12 isolates of the rare Salmonella enterica serovar Adjame were determined by combining Nanopore and Illumina sequence reads. Chromosome sizes ranged from 4,597,011 bp to 4,678,052 bp, and the GC content was 52.3%. A virulent plasmid of 87,433 bp was found in only one isolate.

## ANNOUNCEMENT

Salmonella enterica subsp. *enterica* serovar Adjame was first reported from a clinical case in Cote d’Ivoire in 1967 (1). Although the first case of *S.* Adjame in the United Kingdom was documented in 1993, the first outbreak of *S.* Adjame gastroenteritis was observed in 2017 and was attributed to three distinct clusters based on core genome multilocus sequence typing (cgMLST) and single-nucleotide polymorphism (SNP)-derived phylogenetic analysis (2, 3).

Twelve *S.* Adjame isolates representing the different clusters from the outbreak (*n* = 6) (3) and sporadic cases from the United Kingdom (*n* = 6) (Table 1) were recovered from patients’ stools, typically using xylose lysine deoxycholate (XLD) agar, and were typed by Public Health England. Serotyping was achieved using the Kauffmann-Le Minor procedure, and the serotypes were matched to each sequence type (ST) as previously described (3). Whole-genome sequencing was carried out using an Illumina HiSeq 2500 platform by generating 2 × 100-bp short reads, and isolates were identified as previously described (3). For MinION sequencing, bacteria were grown overnight (16 to 18 h) in 1 ml nutrient broth culture at 37°C in a shaking incubator, and long DNA fragments were extracted using the Fire Monkey/Fire Flower DNA extraction kit (RevoluGen, UK) following the manufacturer’s instructions. Long DNA fragments of adequate quality and quantity as determined with a bioanalyzer (Agilent, Mississauga, ON) and Qubit 2.0 (Life Technologies, Carlsbad, CA), respectively, were used to prepare DNA libraries using the one-dimensional (1D) ligation sequencing kit (SQK-LSK109) and were barcoded (EXP-NBD104 native barcoding expansion kit; Oxford Nanopore Technologies [ONT], UK). Three MinION flow cells (FLO-MIN106 R9.4.1) were loaded with DNA libraries (75 μl) consisting of sequencing buffer (37.5 μl) and loading beads (25.5 μl) added to the DNA (12 μl). Sequencing was carried out over 72 h using MinKNOW software v127.0.0.1, and data were managed with the MinIT device (ONT). Default parameters were used for all software unless otherwise noted. Fast5 reads generated by MinION sequencing were base called and demultiplexed using the high-accuracy Guppy program v3.1.5 followed by adapter trimming using Porechop v0.2.3_seqan2.1.1 (https://github.com/rrwick/Porechop). Trimmed reads were assembled with wtdbg2 software v2.5 (4) and initially polished with Nanopore long reads using the minimap2 tool (5) and SAMtools (6) followed by a second round of polishing by Illumina reads (3) using Pilon v1.23 (7). We also carried out the assembly of the Illumina short reads (3) using Unicycler v0.4.8 (8) to ensure that even smaller plasmids that could have been missed with our long-fragment DNA extraction process used for the MinION sequencing could still be detected as analyzed with the PlasmidFinder software (9). The raw read statistics (numbers, coverages, and average lengths) and genome assembly metrics, including the accession numbers, are shown in Table 1. The 12 *S.* Adjame isolates belonged to two ST, namely, 3929 and 4023, and the eBURST group 421 (3). Nevertheless, the chromosomes had similar sizes ranging from 4,597,011 bp to 4,678,052 bp and similar GC contents of 52.25% to 52.29%. Only one isolate (Illumina SRA number SRR6190984) contained a plasmid, of 87,433 bp, which was found to be similar to the Escherichia coli plasmid pJIE512b (92% coverage and 98.45% identity).

**TABLE 1:** Characteristics of the complete genome sequences of 12 Salmonella enterica subspecies *enterica* Adjame isolates^*a*^

Isolation yr	Cluster or sporadic	Source or reference(s)	ST	Illumina SRA accession no.	Data for MinION reads			Coverage (×)	Genome size (bp)	GC content (%)	Plasmid size (bp)	Assembly accession no.
					No. of reads	Avg read length (bp)	SRA accession no.					
2017	Cluster 1	2, 3	3929	SRR5583198	756,303	8,342	SRR11252159	1,350	4,674,190	52.25		CP049873.1
2017	No cluster	2, 3	3929	SRR6190534	101,869	5,153	SRR11252158	114	4,597,011	52.28		CP049874.1
2017	Cluster 3	3	4023	SRR6190984^*b*^	60,699	6,643	SRR11252167	86	4,673,887	52.26	87,433	CP049875.1, CP049876.1
2017	Cluster 3	3	4023	SRR6190990^*b*^	216,907	4,045	SRR11252166	188	4,673,979	52.26		CP049877.1
2017	Cluster 1	3	3929	SRR6191105	66,491	6,972	SRR11252165	99	4,673,633	52.26		CP049878.1
2008	No cluster	3	3929	SRR6191144	76,939	5,532	SRR11252164	92	4,633,005	52.27		CP049879.1
2017	Cluster 3	3	4023	SRR6191331^*b*^	626,220	4,867	SRR11252163	652	4,678,052	52.26		CP049880.1
2017	No cluster	3	4023	SRR6233875^*b*^	85,451	5,768	SRR11252162	105	4,669,238	52.26		CP054827.1
2017	Cluster 2	3	3929	SRR6233939	80,338	6,056	SRR11252161	104	4,662,031	52.29		CP049881.1
2017	Sporadic	This study	3929	SRR6237069	138,105	5,237	SRR11252160	155	4,662,107	52.29		CP049882.1
2017	Cluster 2	3	3929	SRR6237100	68,051	6,485	SRR11252157	95	4,657,667	52.27		CP049883.1
2017	Sporadic	This study	3929	SRR7426886	606,833	4,971	SRR11252156	647	4,662,216	52.25		CP049884.1

a A representative of strains was selected from a previously described heterogeneous outbreak, describing three clusters (3).

b These are strains from cases that were part of the June to July 2017 outbreak, while the other strains were from the community in the United Kingdom.

### Data availability.

The assembled closed genome sequences of the 12 *Salmonella* Adjame isolates and their MinION sequence raw reads have been deposited in GenBank under the BioProject number PRJNA610035. The accession numbers are provided in Table 1.
